# Acute Airway Compromise Due to Spontaneous Pneumomediastinum in COVID-19 Infection and Subsequent Rapid Formation of Pulmonary Subpleural Bullae: A Case Report

**DOI:** 10.7759/cureus.46011

**Published:** 2023-09-26

**Authors:** Sailesh Partheeban, Kerron Glean, Sharda Boodram, Kristianne Dassrath, Navindra Persad, Prisca Bradshaw, Randall Carvalho, Parasram Ramraj

**Affiliations:** 1 Covid Team, South West Regional Health Authority, San Fernando, TTO; 2 Internal Medicine, South West Regional Health Authority, San Fernando, TTO; 3 Anaesthetics & Intensive Care Unit, South West Regional Health Authority, San Fernando, TTO; 4 Department of Surgery, South West Regional Health Authority, San Fernando, TTO

**Keywords:** case report, bullae, subpleural bleb, airway compromise, covid-19, spontaneous pneumomediastinum

## Abstract

Spontaneous pneumomediastinum (SPM), an increasingly documented complication of COVID-19 infection, usually presents with retrosternal chest pain and dyspnea but can present atypically. In this case, an exceptionally rare presentation could have led to inappropriate management and a poor outcome. Here, a previously healthy 41-year-old Afro-Caribbean male non-smoker presents with acute airway compromise due to SPM. Conservative management proved effective, with anxiolysis to mitigate patient self-induced lung injury (PSILI) and oxygen supplementation via a non-rebreather mask to increase the resolution rate till the patient stabilized over the following days. The sequelae of the lung insult were noted in subsequent imaging, showing the formation of many subpleural bullae. Our case demonstrates the need for a high index of suspicion for pneumomediastinum among teams caring for COVID-19 cases. It also highlights the potential need for follow-up for further research on pulmonary sequelae.

## Introduction

Spontaneous pneumomediastinum (SPM) is emerging as a part of the clinical spectrum of COVID-19 [1]. Herein, we describe a novel case of SPM in COVID-19 leading to acute airway compromise. Note that in our setting during the height of the pandemic (mid-2021) when this case occurred, there existed a “parallel” healthcare system for COVID-19-positive patients that included its own mass casualty style secondary facilities (referred to as “step down facilities”), which also fulfilled a quarantine role, and tertiary centers that would provide specialty & intensive care services. This patient was initially deemed low risk, with mild flu-like symptoms and normal vitals, including oxygen saturation, and was thus under the care of the step-down facility before this clinical event.

## Case presentation

A 41-year-old Afro-Caribbean male non-smoker with no comorbidities, occupational, or recreational exposures to known lung “irritants” was triaged as low severity after presenting with mild COVID-19 symptoms and sent to a step-down facility where he spent his first three days as part of the mass quarantine protocol prior to this episode. Premorbidly, he was physically active with no previous history of respiratory infections.

On day 3 post-admission, the physician was called to review him for sudden onset deterioration with right-sided eyelid swelling, shortness of breath, and dysphagia. The patient’s vitals were a pulse of 120 bpm, a respiratory rate of 38 breaths/min, and SpO2 of 90% on a non-rebreather (NRB) mask with 15 L/min O2 commenced by the nurses. Given that the patient was recently administered a dose of both cefotaxime and guaifenesin with dextromethorphan, treatment for an initial assessment of anaphylactic reaction was commenced, and the patient was then escalated to our tertiary facility.

In all, 500 mcg of IM adrenaline was administered 5 min apart both before and during rapid ambulance transfer with no symptomatic improvement. As we took over the patient from the transfer team, anaphylaxis management was continued with the administration of a third dose of 500 mcg of IM adrenaline, 200 mg of IV hydrocortisone, 10 mg of IV chlorphenamine, and nebulized adrenaline.

At this point, several incongruencies with the initial assessment were noted. The patient had unilateral right-sided palpebral swelling with crepitus to palpation, and subcutaneous emphysema was palpable on the anterior upper thoracic area extending to the neck. The patient had no urticaria or angioedema and was already on IV dexamethasone for the preceding three days. Furthermore, he remained normotensive throughout and had been receiving both the cefotaxime/guaifenesin with dextromethorphan for the last three days with no allergic reactions (note that an antibiotic, steroid, and expectorant were part of the locally chosen management for many symptomatic patients at that point in the pandemic in a bid to prevent progression to needing organ support). The patient’s upper airway demonstrated a Mallampati score of 2 with no swelling of the tongue or oral mucosa. Curiously, a swelling to the soft palate and oropharynx was noted with a displacement of the uvula anteriorly, resulting in it coming to rest on the middle third of the patient’s tongue. Based on these findings, we began to suspect either an underlying pneumothorax or SPM.

A quick literature search found one similar case of airway compromise in SPM in 1996 [2], and taking the lessons from that case into consideration and weighing the risks of intubation (specifically, the risk of potential progression to needing positive pressure mechanical ventilation and air leaking into the already compromised pleura), a conservative anxiolytic strategy was attempted. The intensivist and the surgeon remained present with a difficult airway kit in preparation for failure. Midazolam and fentanyl were administered concurrently with atropine, successfully reducing both his anxiety and complaint of excessive secretions contributing to his distress. The patient was then comfortable, maintaining saturations of 97-99% on NRB with improvement to his pulse of ~100 bpm.

**Figure 1:**
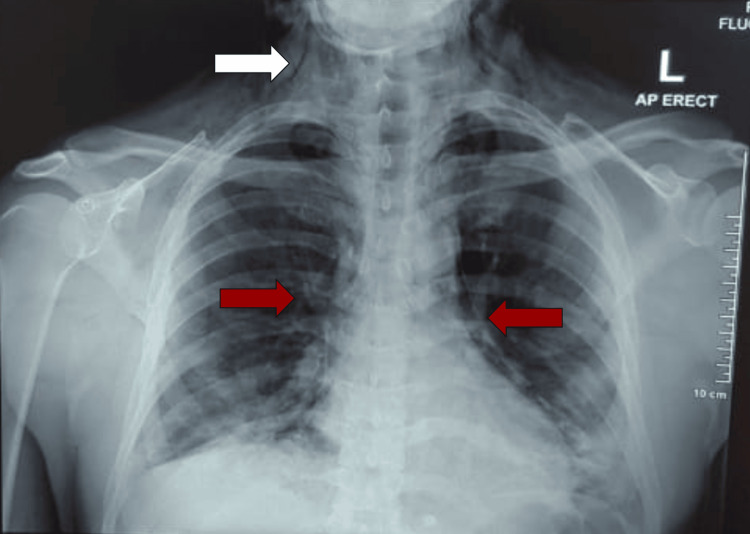
Anterior-posterior chest radiograph showing the curvilinear radiolucent streaks of mediastinal margins (red arrows) and subcutaneous air in the neck (white arrow).

**Figure 2:**
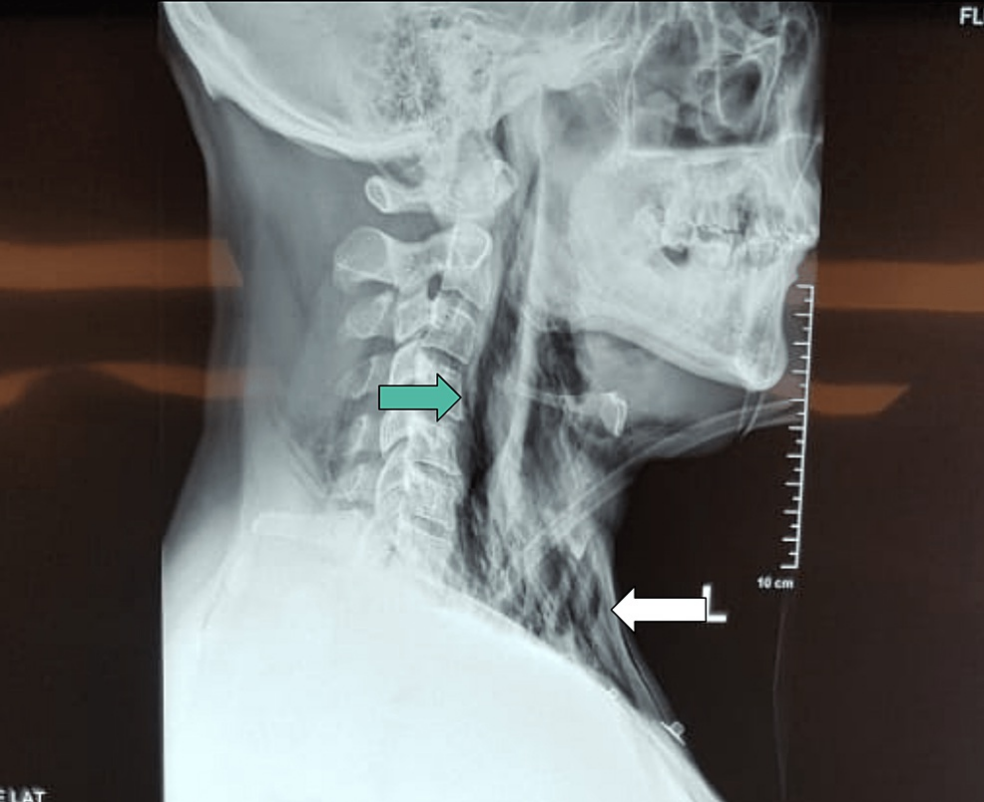
Lateral neck radiograph showing extension dissection of prevertebral soft tissue by air up to nasopharynx (green arrow) and air in anterior cervical tissue (white arrow).

Lung point-of-care ultrasound (POCUS) demonstrated normal lung sliding bilaterally, concordant with anterior-posterior (AP) chest radiograph (CXR) findings (only available later, see Figure 1), which, despite demonstrating a pneumomediastinum, did not suggest pneumothorax or acute respiratory distress syndrome (ARDS). A lateral neck radiograph showed air in the retropharyngeal soft tissues and deep fascial planes of the neck, consistent with the clinical picture (Figure 2). 

As the initial anxiolytic trial wore off, the patient’s clinical features again decompensated and were in keeping with that of “air hunger.” A continuous morphine infusion (dose titrated to response for maximal anxiolysis without potential airway-compromising level of sedation) was commenced and continued for 24 hours to mitigate patient self-induced lung injury (PSILI).

**Figure 3:**
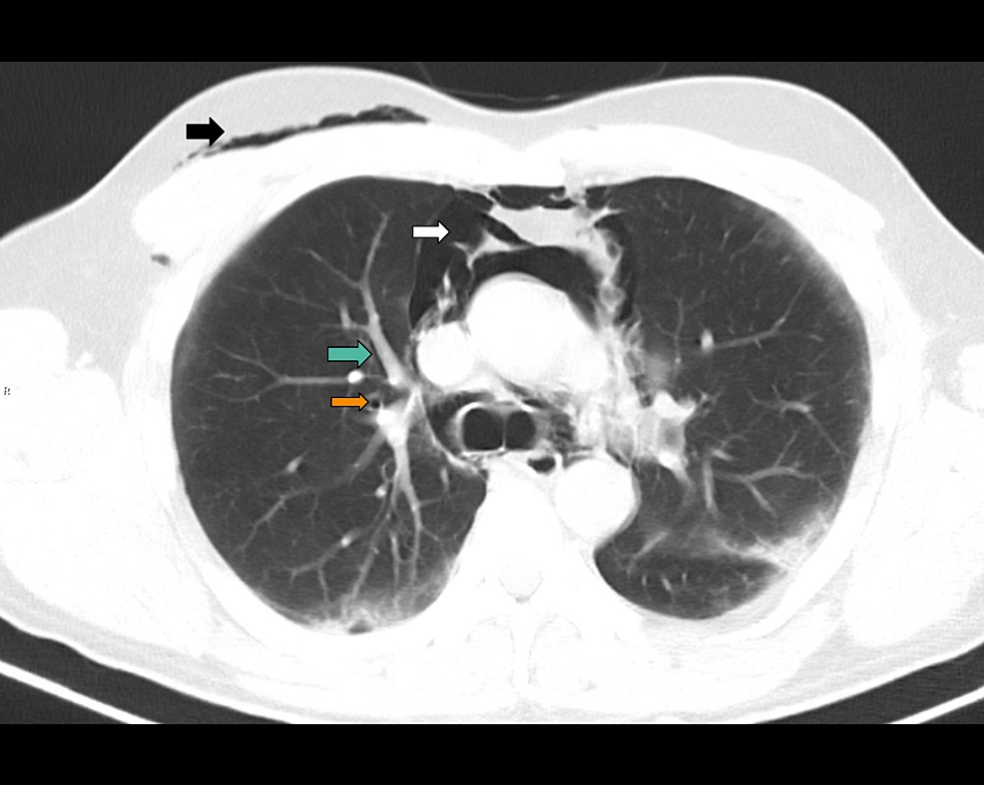
CT scan of the chest from day 3: axial slice showing air around bronchovascular segment aka the Macklin effect (green arrow), pneumomediastinum (white arrow), cystic air space (orange arrow), and subcutaneous emphysema (black arrow).

**Figure 4:**
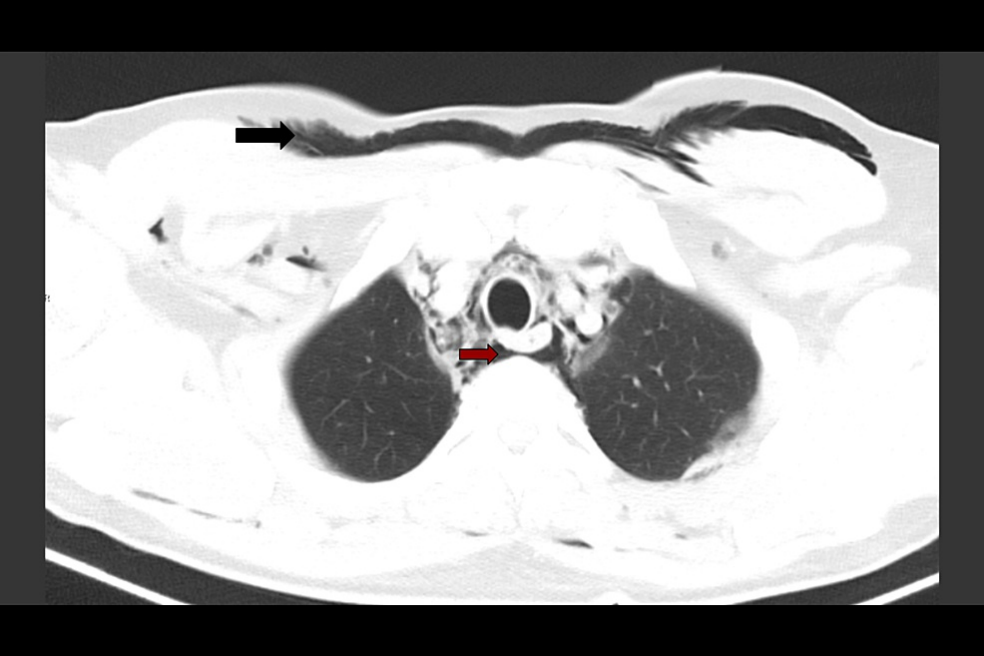
CT scan of chest day 3: axial slice showing air from mediastinum has dissected posteriorly (red arrow) to the esophagus.

**Figure 5:**
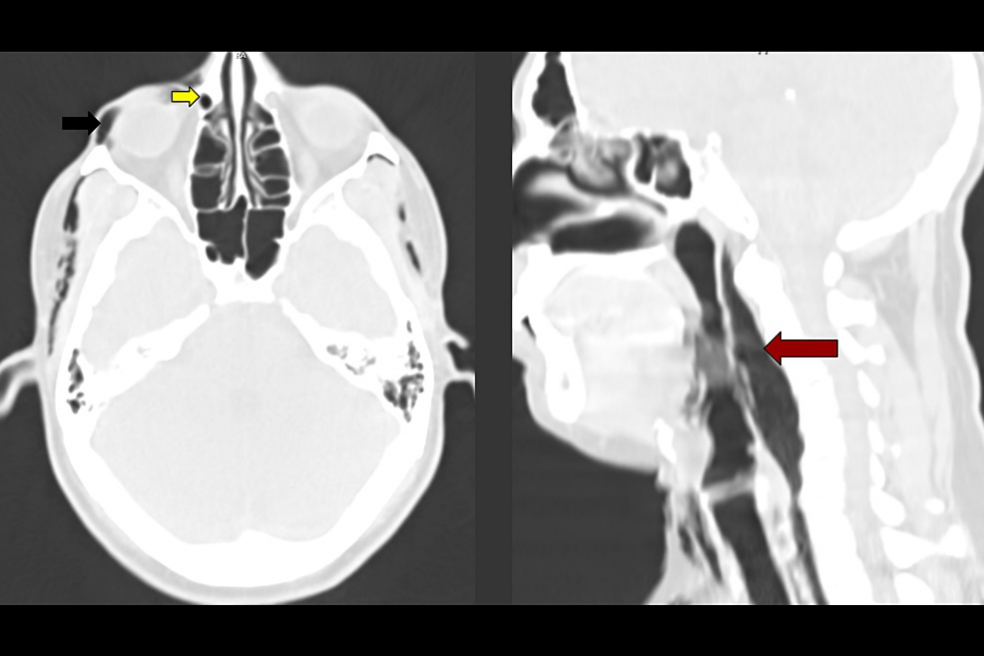
(Left) CT scan of head day 3: axial slice showing right palpebral subcutaneous emphysema (black arrow) and air in the right nasolacrimal duct (yellow arrow). (Right) CT scan of head and neck day 3: sagittal slice showing air dissecting posterior to the pharynx (red arrow) and resultant displacement of posterior structures anteriorly.

A computed tomography (CT) scan of the neck and chest with oral contrast was performed on day 3 of admission (due to scanner unavailability prior to this). By that time, the subcutaneous emphysema had advanced throughout the entirety of the face, but it had improved around the right eyelids, which allowed the patient to use his right eye again. Neither tracheal injury nor oral contrast extravasation was evident on this CT. However, ground glass opacities, evidence of the “Macklin effect” (alveolar rupture leading to air dissecting along the bronchovascular sheaths and then into the mediastinum), and a few scattered cystic air spaces in the lung parenchyma were seen (Figure 3). Most interestingly, the findings on the CT scan explained and documented the air dissecting posterior to the esophagus (Figure 4), all the way posterior to the pharynx and into the right nasolacrimal duct (Figure 5).

He remained stable over the following days despite intermittent desaturation episodes, which resolved spontaneously. He had lung POCUS and CXRs daily during this time. By day 6, there was clinical resolution of the subcutaneous emphysema to his face, neck, right palpebral tissue, and chest wall.

**Figure 6:**
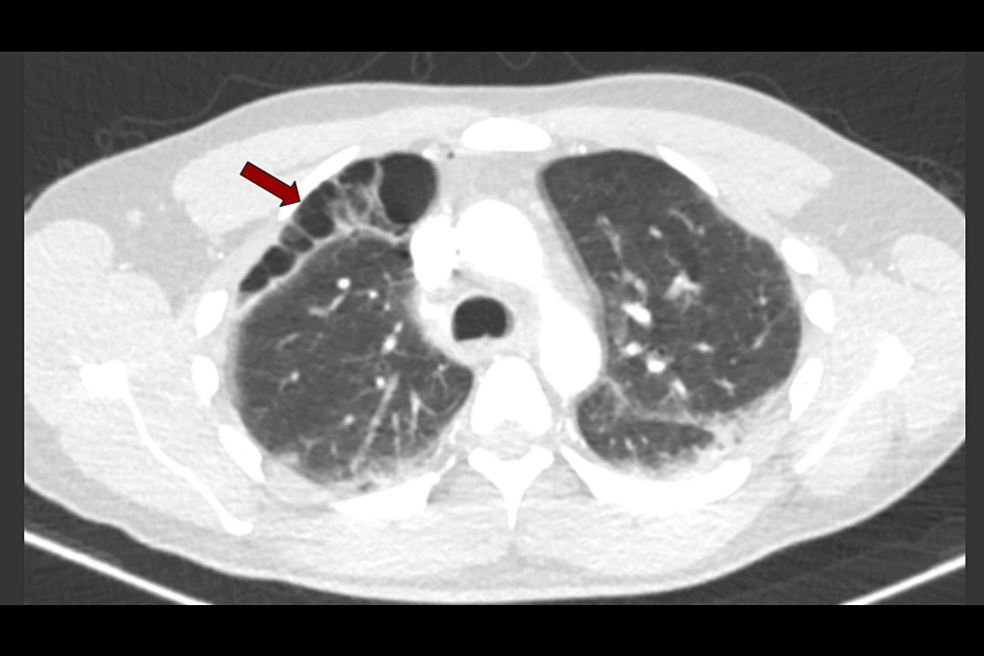
CTPA day 9: axial slice showing the location of subpleural bullae anteriorly (red arrow) CTPA, computed tomography pulmonary angiogram

**Figure 7:**
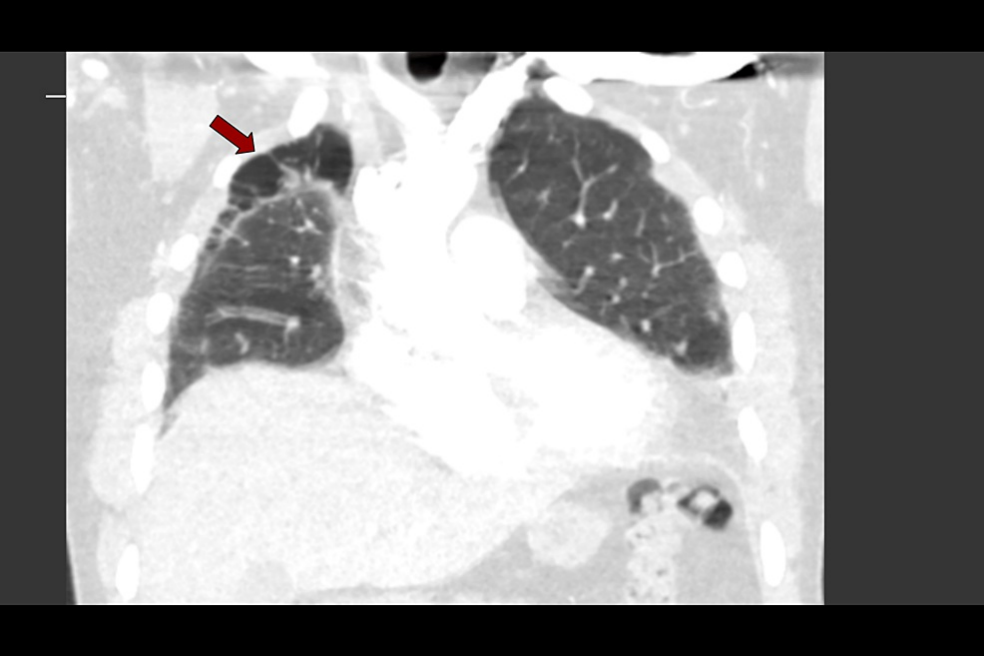
CTPA day 9: coronal slice showing apical location of subpleural bullae (red arrow), and presence of small cystic air spaces can also be seen scattered elsewhere in the lung CTPA, computed tomography pulmonary angiogram

**Figure 8:**
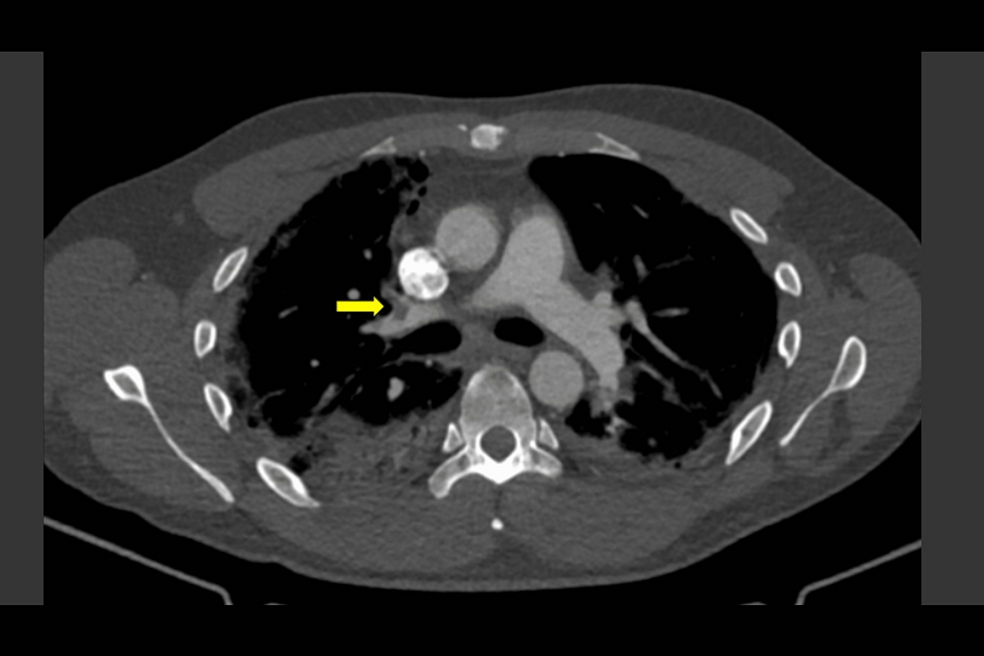
CTPA day 9: axial slice in the bone window showing a right segmental pulmonary embolism (yellow arrow) in situ CTPA, computed tomography pulmonary angiogram

On day 8 following admission, he had a sudden onset of profound hypotension and tachycardia with reduced SpO2, which spontaneously resolved within the hour. He had no evidence of recurrence of subcutaneous emphysema or hyperresonant percussion. A CT pulmonary angiogram (CTPA) was done, which showed apical subpleural blebs (Figures 6, 7) that were not present in the initial CT scan and a right segmental pulmonary embolism (PE) (Figure 8), for which he was started on rivaroxaban 15 mg PO twice daily.

The patient was subsequently returned to a step-down facility after spending 12 days in the tertiary hospital and referred to the medical outpatient clinic for continued follow-up in lieu of unavailable subspecialty respiratory medicine services.

## Discussion

At the time of this case’s presentation, there was only one relevant publication [2] that we could use as a reference to reaffirm our suspicions that this rare presentation was, in fact, even possible and as dangerous as we suspected and change our treatment pathway. SPM has been a recognized clinical manifestation in patients with COVID-19, affecting up to approximately two in 1000 hospitalized patients, the majority of which are receiving positive pressure airway support and a minority of which occur in unassisted breathing [3]. Ours is one such case where the onset of SPM has occurred in an unassisted breathing patient, initially triaged as a mild illness but requiring hospital admission and quarantine and was thus warded in a secondary/step-down facility for lower-risk patients when his dramatic presentation ensued.

SPM can often be challenging to diagnose due to the spectrum of presentations and complications associated with COVID-19. Notably, the “typical” sudden onset retrosternal chest pain was not present here; however, subcutaneous emphysema, which may be detected in 70% of cases [4], was. Despite this, the patient was initially treated as an anaphylactic reaction due to the airway compromise, of which SPM is not one of the foremost causes (the abovementioned 1996 case being the only such record we could find). Thankfully, the tertiary care team explored alternative diagnoses by noting the multiple incongruencies.

Here, a readily available portable AP CXR proved invaluable, suggesting SPM while lung POCUS allowed the rapid exclusion of pneumothorax. At its worst, the emphysema on the anterior chest wall completely prevented the conduction of ultrasound, so we could only check for a “sliding sign” posteriorly. CT chest is the definitive assessment, helping to assess the underlying parenchyma while ruling out more dangerous causes, such as tracheal and esophageal (when paired with oral contrast) injury [5-7]. Occasionally, bronchoscopy or laryngoscopy may be helpful to rule out the presence of tracheal tears, especially if this is suggested on CT; however, as this is less likely in the setting of an atraumatic and uninstrumented airway, it was foregone.

Our patient proved highly atypical; he was, prior to this episode, a comfortable patient in nil respiratory distress with mild illness and, by all accords, would not have been the candidate to experience a complication associated with barotrauma. His initial CT findings give insight into both this and the cause of his airway compromise. Firstly, there are a few scattered cystic air spaces seen in the lungs, which can be thought of as the early stage of the same process that led to the formation of his subpleural blebs later, a macroscopic consequence of the theorized “diffuse alveolar rupture” [8]. These likely proved to be a point of weakness, and one of these either ruptured spontaneously or with help from a bout of coughing, providing a transient intolerable pressure change to that tissue [9]. Secondly, the Macklin effect [10,11] is demonstrated here, so the air entering through the ruptured “sack” is dissected along the plane of the peri-bronchovascular tree (highlighted in Figure 3 by green arrow), following it into the mediastinum. Air can be seen dissecting the paratracheal soft tissues ascending in the superior mediastinum, then through the entirety of the pharynx, and up through the soft tissues in the left nasolacrimal duct, finally reaching the left palpebral subcutaneous tissue. This anatomical pathway of ascent anteriorly displaced these posterior structures, thus narrowing the supraglottic inlet. Our initial visual inspection was concordant with this, with the anteriorly displaced uvula and swelling to the oropharynx, sparing the anterior structures.

An alternative possibility is the emphysema arising up in the neck anteriorly that led to venous congestion and edema, quite similar to the airway compromise seen in a strap hematoma as a complication post-thyroid surgery. However, we postulate this is less likely the case with our patient due to the timing; on presentation, there was no emphysema to the neck or facial tissues. This was a later development noted to be in continuity with, and gradually ascending from, the anterior chest subcutaneous emphysema, and the patient’s airway compromise had improved by this point. Had CT been available at the point of admission, we could have been more conclusive.

Usually a relatively benign entity, patients with SPM and not on positive pressure ventilation pre-COVID-19 were generally treated conservatively with bed rest, antitussives, and oxygen therapy to aid in reabsorption of free air with analgesia or anxiolysis where required [12]. Complicated cases are known to benefit from surgical input, especially in the case of the rare malignant pneumomediastinum, which may cause tamponade and tension and be associated with an ongoing air leak, but this proved unnecessary in our case.

We tailored our conservative approach by identifying the two factors, both potential causes of SPM in and of themselves, that could worsen the clinical presentation.

The first is PSILI, a form of volutrauma. At symptom onset, our patient was profoundly dyspneic, displaying signs consistent with “air hunger.” Initially, his increased drive, being a response to hypoxia from the airway compromise, was then likely potentiated by the adrenaline therapy instituted for anaphylaxis. PSILI is brought about by extreme inspiratory efforts, which cause global and local airway distention, notably two things that adrenaline also facilitates. This can lead to changes in transpulmonary and transvascular pressures and even cause diaphragmatic injury [13]. In our case, it could worsen the existing damage forcing enough air past to cause an even smaller airway inlet or perhaps even tamponading vessels/heart/trachea with the dreaded “malignant pneumomediastinum,” leading to cardiac tamponade. As a result, morphine became our drug of choice for our intravenous continuous anxiolytic strategy. With its role in reducing respiratory drive, we immediately noticed the cessation of air hunger behavior, and it effectively reduced minute ventilation without any compromise to oxygenation.

The second factor is mechanical ventilation. The fact that a positive pressure strategy would further our patient’s morbidity is strongly suggested in recent studies, showing mortality in excess of 70% with ventilator-dependent cases of COVID-19 pneumonia [14,15]. Even more so once we note the rapidity with which our patient developed subpleural blebs. As part of the treatment plan, it was decided that should we be forced to intubate, we would use a spontaneous ventilator mode with minimal positive pressure exposure. Notably, the index case in 1996 did end up intubated and then was extubated after two days. That case predates COVID-19 and, in the absence of the diffuse alveolar injury of the inflammatory process involved there, was perhaps a truly viable option; however, there is no reference in that case report of what ventilation settings are used.

The rapid progression of lung disease from cystic air spaces seen on initial CT to the apical subpleural blebs is remarkable, and we wonder whether this is illustrative of the role of neutrophils in degrading lung elastance [16]. The severity at the right apical location may even reflect the shunting of these neutrophils to the well-perfused apex in light of the segmental vessel PE in the lower lung and whatever degree of basal atelectasis. Many intriguing clinical questions arise, such as the potential role of an IL-6 inhibitor (such as tocilizumab, which is used in select severe COVID-19 cases) in reducing disease progression. Another concern in a possible subpopulation of COVID-19 patients with SPM and bullae would be the possible progression to spontaneous pneumothoraces, with associated long-term complications requiring thoracic surgical intervention. A future study by a large-volume respiratory service in a higher resource setting will likely bring interesting results in this field.

## Conclusions

This rare presentation of SPM with acute airway compromise was eventually explained anatomically by the imaging findings, and it reaffirmed the need for clinicians to have a high index of suspicion and follow up on any incongruencies. The subsequent degeneration of our patient's right lung apex into subpleural bullae in the absence of comorbidities poses clinical questions. One of which is whether a currently asymptomatic group of patients now has this pathology and might be at risk of SPM or pneumothorax. We hope that this case report assists clinicians who are presented with such an outlier and may be of interest to those in more high-resource healthcare settings operating specialist respiratory units that follow up on these patients.
